# The influence of lateral dominance on bilateral performance in alpine skiers

**DOI:** 10.3389/fspor.2026.1717506

**Published:** 2026-01-30

**Authors:** Yunfei Huang, Yiquan Yin

**Affiliations:** 1 Sports Coaching College, Beijing Sport University, Beijing, China; 2 Department of China Skiing and Ice Sport College, Beijing Sport University, Beijing, China

**Keywords:** alpine skiing, bilateral asymmetry, eye dominance, lateral dominance, leg dominance

## Abstract

**Purpose:**

This study investigated the impact of lateral dominance (eye/leg dominance and ipsilateral/crossed dominance patterns) on bilateral turning performance in alpine skiers, analyzing its mechanistic role across different skiing phases to provide theoretical support for training optimization.

**Methods:**

Twenty-two alpine skiers (age: 23.14 ± 1.75 years; national level 1 or above) performed slalom tests on an indoor ski simulator (slope: 20°; speed: 27.3 km/h). 4 K cameras recorded kinematics and time metrics during initiation phase, and turning phases. Post-test, eye dominance was determined via hole-in-card and electrooculography tests, while leg dominance was assessed through single-leg vertical jumps. Participants were categorized into ipsilateral/crossed dominance groups based on eye-leg combinations. Mixed linear models analyzed within-group differences across phases.

**Results:**

During initiation phase, dominant eye-turn consistency showed a significant effect, where turns towards the non-dominant side yielded superior performance (*β* = −0.010, *P* = 0.015). In the completion phase, interaction effects indicated that ipsilateral dominance yielded superior performance specifically when turns were consistent with the dominant side *P* = 0.021, partial *η*² = 0.021; *P* < 0.001, partial *η*² = 0.027). Cross-dominant skiers demonstrated the poorest performance in single-turn metrics (*P* < 0.05), potentially due to interhemispheric integration delays. Laterality influences slalom performance through visuomotor coupling mechanisms, with ipsilateral dominance showing neural efficiency advantages during initiation.

**Conclusion:**

Coaches should incorporate dual-task training to improve visual-motor coordination and bilateral symmetry, thereby mitigating technical asymmetries and enhancing competitive outcomes.

## 1 Introduction

In competitive sport, In competitive sports, the precision and efficiency of movement are key factors for an athlete's performance. Alpine skiing is a highly demanding snow sport that places extreme demands on an athlete's performance capabilities and physical fitness. In the slalom event, the course slope typically ranges from 25° to 65°. Skiers are required to pass through tightly set gates at speeds of up to 80 km/h while performing complex and technically demanding turns (1, 2). This process, skiers need strong lower-body strength and excellent dynamic balance to handle the intense impacts and centrifugal forces from high-speed skiing (3). At the same time, they must quickly process complex visual information to accurately plan the best racing line (4). This close coordination between vision and lower-body movement is essential for precise body control (5). In alpine skiing competitions, victory is often decided by milliseconds. For instance, the gap between gold and silver can be as small as one-thousandth of a second. In such a demanding competitive environment, even minor performance deviations, particularly bilateral asymmetry in movements, can significantly affect the final result (1).

Undoubtedly, a key goal of systematic training for athletes is to achieve consistent performance on both sides of the body. This allows them to ski with minimal energy expenditure and optimal efficiency. In sports science, bilateral asymmetry refers to measurable differences between the left and right sides in functional outputs like strength, completion time, or movement patterns (6, 7). During training and competition, athletes often show this asymmetry. For example, one side may have faster and more precise turns, while the other side is slower. In some cases, one side may exhibit excessive body rotation, while the other remains more stable. This bilateral asymmetry can lead to decreased performance, inconsistent results, and a higher risk of injury (8, 9).

While the causes of asymmetry are complex, previous research has primarily focused on differences in lower limb muscle strength or joint kinematics (9, 10). Specifically in alpine skiing, studies focusing on biomechanics have found significant differences in technical movements and Ground Reaction Forces (GRF) between left and right turns in elite skiers (11). Such asymmetry in force distribution and body posture can result in higher GRF on one side, thereby reducing skiing efficiency. Additionally, research indicates that a difference of approximately 10% in functional output between the left and right legs leads to uneven GRF distribution during turns (8).However, in practical training, continuous monitoring with biomechanical instruments is difficult to implement constantly. Instead, coaches primarily rely on subjective movement guidance (12, 13). This qualitative approach often targets visible technical flaws but may fail to resolve the underlying causes. Consequently, bilateral performance asymmetry remains a persistent issue among athletes. Most existing research focuses on these biomechanical parameters. However, it has largely overlooked the role of lateral dominance, a factor that may also contribute to bilateral performance differences (14, 15).

Lateral dominance, or laterality, refers to the preferential use of one side of the body. This concept typically involves the hand, foot, eye, or ear. It is not a single trait but a complex neuropsychological system (14, 16, 17). This system comprises several domains, including handedness, footedness, ocular dominance, and auditory dominance (18). Some studies suggest that lateral dominance or laterality can influence motor performance in sport specific tasks (14). In many investigations, the term lateral preference is used interchangeably with lateral dominance (19, 20). While definitions and terminology vary across studies, the underlying idea is consistent: individual side-bias or limb dominance may affect motor coordination and lead to inter limb performance differences. In other sports, lateral dominance significantly influences performance efficiency. Specifically, cross-lateral dominance opposite eye-hand/foot enhances visual tracking and force transmission in dynamic tasks like tennis serving and baseball batting (21–23). In contrast, ipsilateral dominance favors static precision tasks like soccer penalty kicks, whereas cross-lateral patterns can create neural signal conflicts, reducing coordination and scoring efficiency in sports like fencing (9).

The primary aim of this study was to investigate the influence of lateral dominance on bilateral skiing performance, specifically examining how leg dominance, eye dominance, and their interactions (same-side vs. crossed dominance) affect performance differences in skiing turns. Based on the demands of slalom skiing, we proposed three hypotheses. First, regarding eye dominance, we hypothesized that turns towards the non-dominant eye side would result in better performance (shorter time). Second, for leg dominance, we hypothesized that turns towards the dominant leg side would show improved performance due to greater strength and stability. Finally, regarding the interaction effect, we hypothesized that same-side dominance profiles (e.g., right eye, right leg) would lead to better performance in skiing turns. For example, a skier with same-side dominance (right eye, right leg) is expected to perform optimally when turning towards the dominant leg side (right turn), as this direction combines the mechanical advantage of the dominant leg with the visual advantage of turning towards the dominant eye.

## 2 Methods

### 2.1 Participants

This study recruited 22 national-level alpine skiers (15 males, 7 females), all certified as Class I or higher athletes (China's national athlete grading system) with ≥4 years of training experience. Participant demographics are detailed in (Table 1). Inclusion criteria included no organic visual impairments, myopia, or lower limb sports injuries within the past 6 months; no clinically significant musculoskeletal asymmetries, such as pathological strength imbalances caused by injury; and the capability to continuously perform high-intensity skiing tasks on simulators with movements complying with standard skiing techniques. Prior to testing, all participants were informed of experimental procedures and potential risks. Written informed consent was obtained voluntarily. The study protocol was approved by the Joint Health Research Ethics Board of Beijing Sport University (Approval No. 2025349H), adhering to the principles of the Declaration of Helsinki.

**Table 1:** Participant characteristics.

Name	Age	Gender	Sport grade	Training experience
Liu X	21	Female	National Master	5
Zhang XX	21	Female	National Master	6
Zhu XX	23	Male	National Master	4
Huang XX	22	Female	National Master	7
Jin X	22	Male	National Master	8
Dong X	24	Male	National Master	8
Jiang X	26	Male	National Master	4
Zhu XX	21	Female	National level	4
Liu XX	21	Female	National level	4
Bai XX	22	Male	National level	5
Zhao XX	21	Male	National level	5
Yu X	22	Male	National level	6
LI X	24	Male	National level	4
Liu X	22	Male	National level	8
Zhang X	21	Male	National level	6
Sheng X	21	Male	National level	6
Yu XX	22	Male	National level	4
Liu XX	21	Male	National level	4
Cai X	24	Male	National level	10
Chen XX	23	Female	National level	3
Zhang XX	23	Male	National level	9
Wang XX	21	Female	National level	7

### 2.2 Testing equipment and procedures

#### 2.2.1 General testing protocol

**Figure 1:**
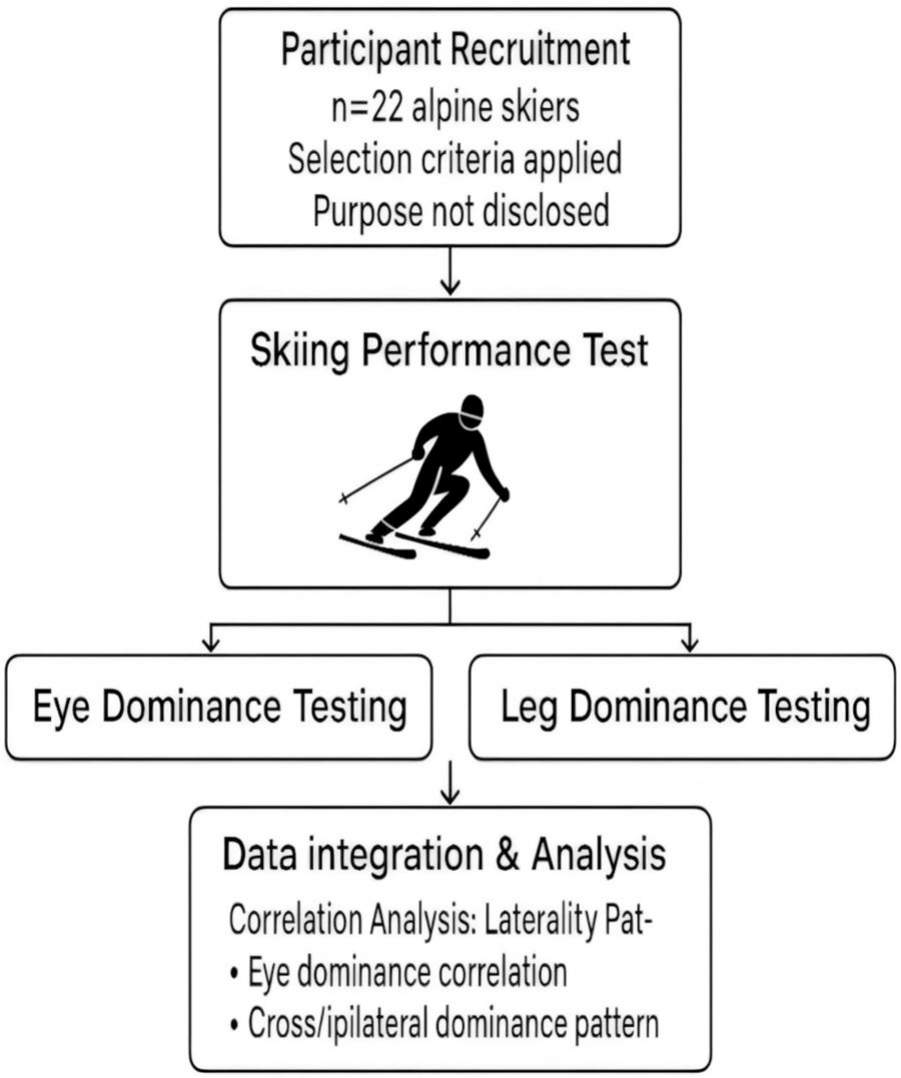
Experimental testing protocol.

To control variables, participants were only informed of the testing requirements prior to the experiment, with no disclosure of the study's specific objectives. Data collection was followed by dominant eye and leg measurements (see Figure 1).

#### 2.2.2 Skiing test

**Figure 2:**
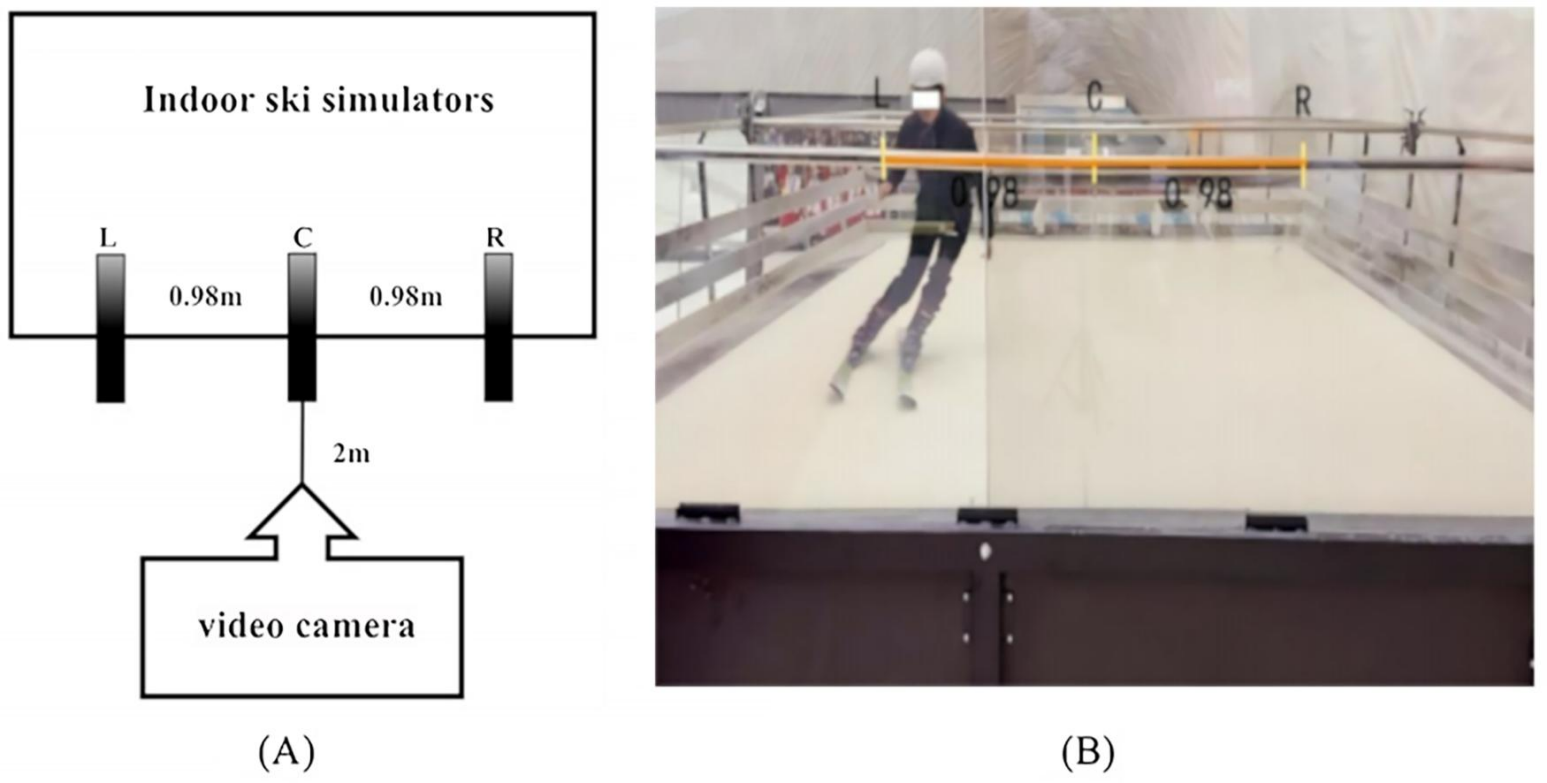
Camera placement and slalom gate configuration. **(A)** Schematic diagram with specific planar measurements: Point C represents the skiing start/end point and central turning point; R and L denote the right and left turning points, respectively. CL = CR = 0.98 m, LR = 1.96 m. The camera was positioned 2 m above the ground and 2 m away from the central point C. **(B)** Actual camera setup during field testing.

This study employed a cross-sectional experimental design to investigate the relationship between eye-leg dominance patterns and alpine skiing performance. Tests were conducted on the Aisinuo ASN-XL indoor skiing simulator (slope: 20 ± 0.5°, speed: 27.3 km/h ± 0.5 km/h). Three reflective marker gates were arranged linearly to define turning points (CL = C = 0.98 m, LR = 1.96 m), including a central starting point (C) and left/right turning points (L/R). A 4 K camera (120 Hz) was positioned 2 m from the centerline at a height of 2 m to record all trials (see Figure 2).

Participants began at the central point and executed 30 gate-passing runs (15 left/right turns each). No external feedback was provided during testing to indicate successful gate clearance. All trials were recorded and analyzed using Kinovea (https://www.kinovea.org) motion analysis software to extract precise phase-specific timing data (24, 25).

#### 2.2.3 Dominant eye assessment

This study utilized two authoritative and complementary methods to determine participants' dominant eyes: the Hole-in-Card Test and Electrooculography (EOG).

The Hole-in-Card Test, a classic and widely adopted method, was conducted as follows: Participants held a 20 × 30 cm cardboard (with a central 3 cm diameter hole) with both hands, extending their arms to maintain a 40 cm distance from the face. They fixated on a target marker (a bold black cross at 1.7 m height) 6 meters away through the hole while keeping their head and body stationary. The examiner alternately covered the left and right eyes. If the target marker disappeared from view when one eye was covered, the uncovered eye was identified as dominant. To ensure reliability, each participant repeated the test 3 times, with a valid result requiring at least 2 consistent outcomes (26–28).

As a supplementary method, Electrooculography (EOG) was employed for objective assessment. Tests were conducted in a light-controlled laboratory: Participants sat 60 cm from a display screen with their heads stabilized on a chin rest to minimize motion artifacts. Surface electrodes were attached around the eyes to record potential changes during eye movements. The protocol included three phases: ① Baseline recording (fixation on a central point); ② Saccadic test (rapid shifts between left/right targets); ③ Smooth pursuit test (tracking a moving object). Dominance was determined by analyzing latency, velocity, and accuracy of eye movements (14). The combined results classified participants' dominant eyes (left/right) for subsequent analysis.

#### 2.2.4 Dominant leg assessment

This study determined participants' dominant legs through the Single-Leg Vertical Jump Test, conducted on a force platform (Kistler Type 9287CA, Kistler Group, Switzerland) connected to a data acquisition system. The system recorded ground reaction forces (GRF) and related kinetic parameters in real time, ensuring precise analysis. Prior to testing, a 5–10 min warm-up (including light aerobic exercises and lower-limb stretches) was performed to activate muscles and reduce injury risks (29, 30). Participants wore athletic shoes and stood at the center of the platform with hands placed naturally on their hips.

During testing, participants stood on one leg (right leg first, followed by the left), stabilized their posture, and jumped vertically as rapidly and forcefully as possible before landing back on the platform. The force platform captured real-time data, including peak vertical GRF, impulse, and flight time (the core parameter for calculating vertical jump height). Each leg underwent three valid trials; data from unstable landings or platform deviations were excluded. The average vertical jump height from three valid trials was recorded as the final score for each leg. The leg with the higher score was identified as the dominant leg.

### 2.3 Operational definition

#### 2.3.1 Ipsilateral and crossed dominance

Ipsilateral Dominance: Determined based on the participant's dominant leg and dominant eye. If both were on the same side of the body (e.g., right leg and right eye dominant), they were classified as ipsilateral dominance.

Crossed Dominance: If the dominant leg and dominant eye were on opposite sides (e.g., right leg dominant but left eye dominant), they were classified as crossed dominance.

Among the 22 participants in this study, 11 exhibited ipsilateral dominance (8 males, 3 females) and 11 exhibited crossed dominance (7 males, 4 females).

#### 2.3.2 Definition and extraction of skiing data metrics

**Figure 3:**
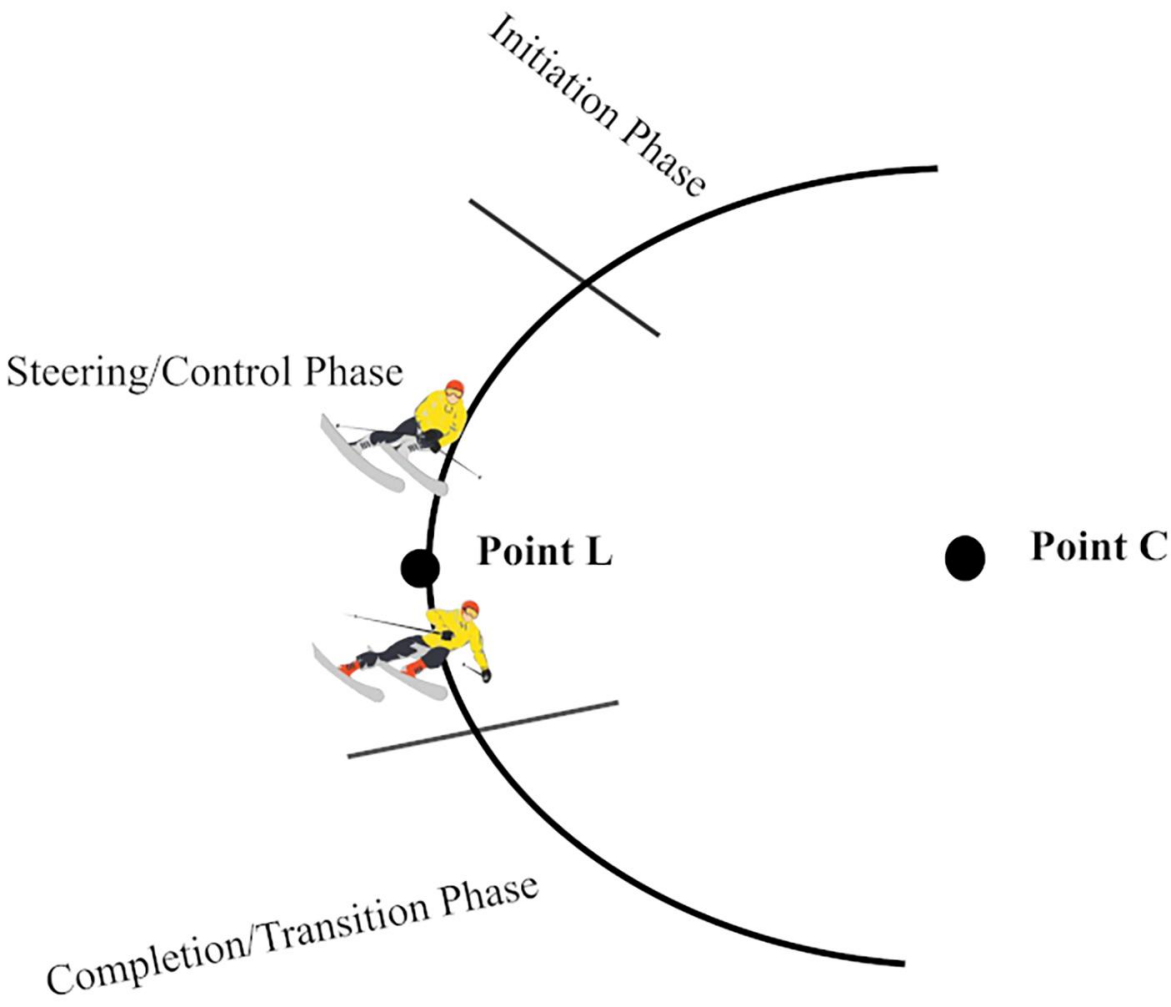
Schematic diagram of the three skiing phases in alpine skiing.

**Figure 4:**
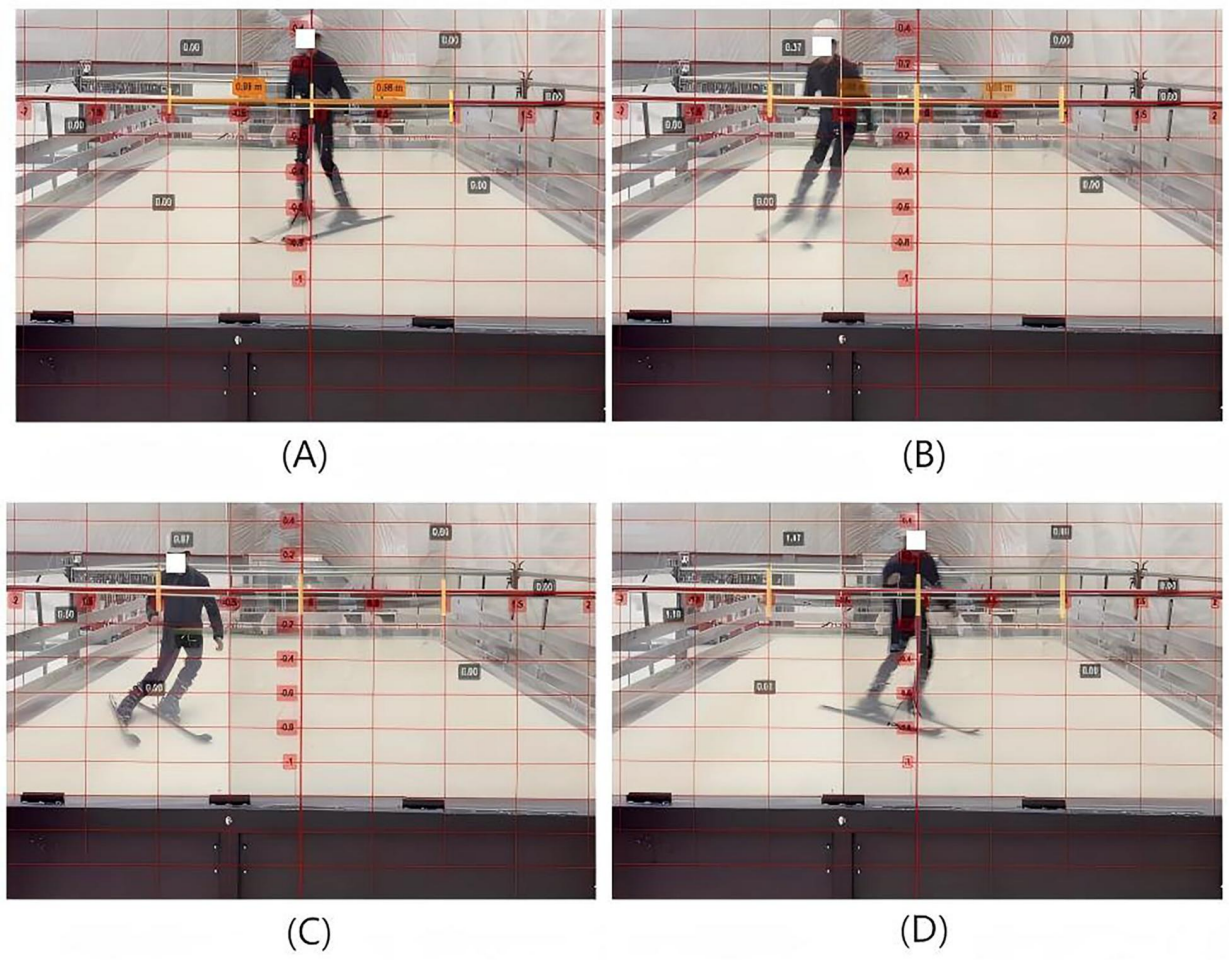
Illustrates parameter extraction for alpine skiing phases. **(A)** Start of skiing (beginning of initiation phase); **(B)** End of initiation phase and start of Steering Phase; **(C)** End of Steering Phase and start of Completion Phase; **(D)** End of Completion Phase (concluding a full turn).

According to literature (31–33), the alpine skiing gate-passing technique is commonly divided into three phases based on the majority of researchers' classifications. This study adopts this tri-phase division and analyzes skiing distance (see Figure 3 and Figure 4). The specific phases are as follows:

1. initiation phase: From the start of the turn until the ski edge contacts the snow surface, i.e., from the central point (Point c) to the leading edge of the turning point.
2. Steering/Control Phase: From full edge contact with the snow to the beginning of ski flattening.
3. Completion/Transition Phase: From the initial flattening of the ski edge to the start of the next turn.

### 2.4 Statistical analysis

All statistical analyzes were performed using SPSS Statistics 30.0. The study included 22 athletes (11 with crossed dominance, 11 with ipsilateral dominance), each completing 30 trials (15 per turning direction), resulting in 660 total observations. Athlete ID was incorporated as a random effect in the model to control for inter-individual variability (34).

A linear mixed-effects model (LMM) was employed to analyze the impact of lateral dominance on alpine skiing performance across different phases (35). The models included lateral dominance type (crossed vs. ipsilateral), dominant eye-turn direction consistency, and dominant leg-turn direction consistency as fixed factors. Turn direction (left vs. right) was not directly included as a fixed factor but was implicitly represented through the “consistency” variable (e.g., consistency between dominant eye/leg and turn direction). No additional covariates, such as individual characteristics (e.g., age or experience level), were included in the model, as our primary focus was on lateral dominance (dominant eye, dominant leg, and crossed/ipsilateral dominance) and its direct effects on performance.

Separate models were constructed for dominant eye and dominant leg to examine their respective effects on skiing performance. Dependent variables included four performance metrics: initiation phase time, Steering phase time, Completion phase time, Total one-turn time Model assumptions were verified through: (1) Normality of residuals (Shapiro–Wilk test). (2) Homoscedasticity of residuals (Levene's test). (3) Normality of random effects (36).

Two binary lateral dominance-related predictors were defined: "Dominant Eye-Turn Direction Consistency": Coded as “consistent" (e.g., right-eye-dominant athlete turning right) or “inconsistent" (e.g., right-eye-dominant athlete turning left). "Dominant Leg-Turn Direction Consistency": Coded identically to the eye model Lateral Dominance Pattern: Classified as crossed dominance (e.g., right-eye-dominant + left-leg-dominant) or ipsilateral dominance (e.g., right-eye-dominant + right-leg-dominant).

Preliminary analysis revealed strong collinearity between eye and leg consistency predictors. To avoid multicollinearity and ensure parameter stability, separate models were fitted for each performance metric:

Model 1 (Dominant Eye Effect Model):

$$\begin{array}{rl} Y\_ij= & \beta \_0+\beta \_1\;(Crossed/Ipsilateral)\\ & +\beta \_2\;(Eye-Turn\;Consistency)+\beta \_3\;(Interaction)+u\_i\\ & +\varepsilon \_ij \end{array}$$

Model 2 (Dominant Leg Effect Model):

$$\begin{array}{rl} Yij= & \beta 0+\beta 1\;(Crossed/Ipsilateral\;Dominance)\\ & +\beta 2\;(Dominant\;Leg-Turn\;Direction\;\;Consistency)\\ & +\beta 3\;(Crossed/Ipsilateral\;Dominant\times Dominant\;Leg\\ & -Turn\;Direction\;Consistency)+ui+\varepsilon ij \end{array}$$

Where *Yij* represents the performance measure for the jth measurement of the ith athlete, *β* denotes the fixed-effect parameters, *ui* represents the random intercept for athlete *i*, and *εij* represents the residual term. It is assumed that *ui* ∼ *N* (0, *σ*²*u*), *εij* ∼ *N* (0, *σ*²*ε*), and that the random effects and residual terms are mutually independent (36).

Parameters were estimated using the Restricted Maximum Likelihood (REML) method, which provides less biased variance component estimates in small-sample scenarios (34). Significance tests for fixed effects were conducted using *F*-tests, while variance components for random effects were tested using Wald *Z*-tests.

The significance threshold was set at α = 0.05. For significant interactions, estimated marginal means (EMMs) with Bonferroni-adjusted pairwise comparisons were conducted to control family-wise error rates. Simple effects analysis interpreted interaction patterns, clarifying effect differences across lateral dominance combinations.

## 3 Results

**Figure 5:**
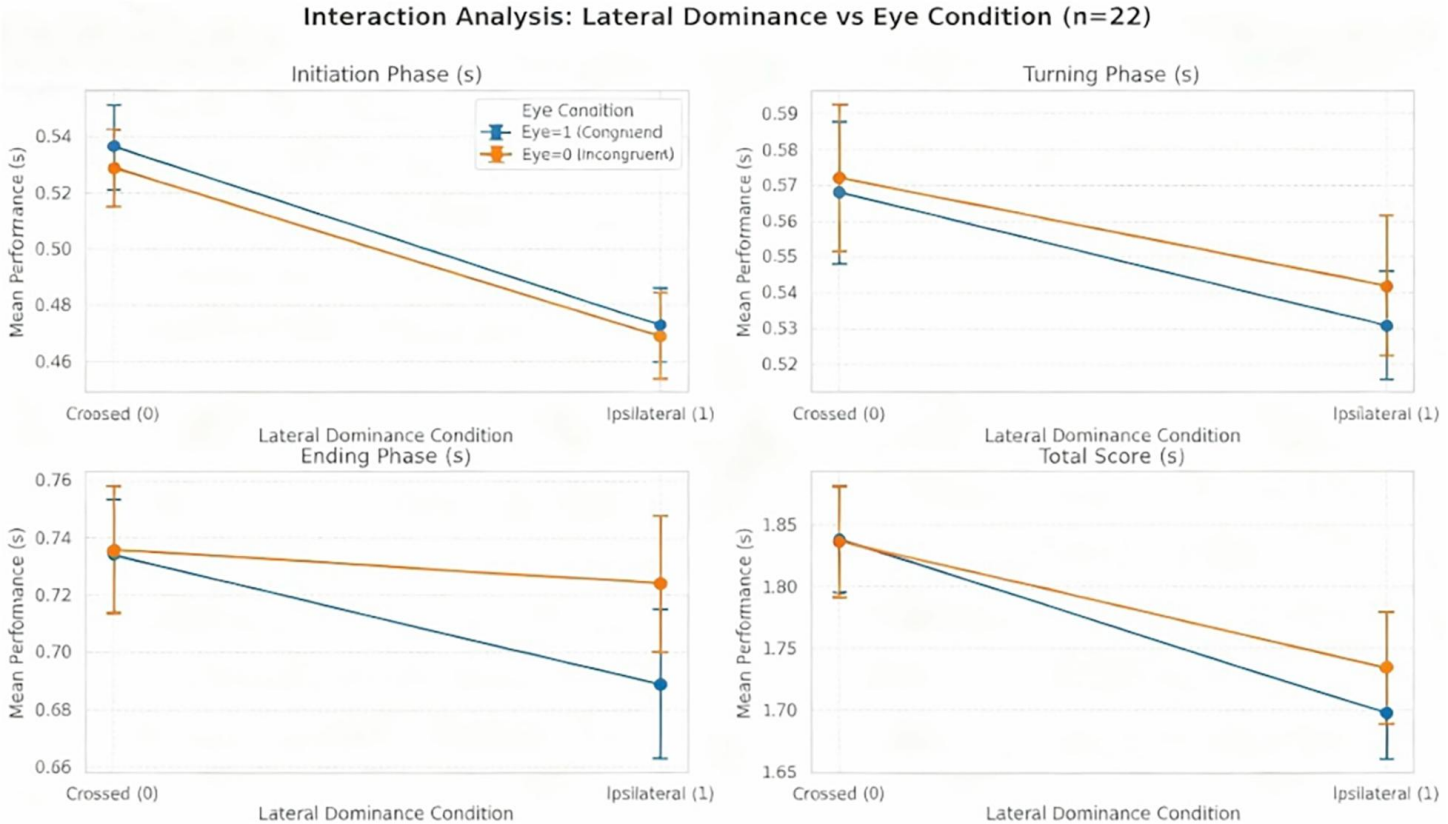
Interaction between lateral dominance and eye congruence on performance metrics. Performance times (mean ± SEM, *n* = 22) during **(A)** initiation phase, **(B)** turning phase, **(C)** ending phase, and **(D)** total performance. Blue line: congruent eye condition (dominant eye aligned with movement direction). Orange line: incongruent eye condition. Significant interaction patterns were observed where congruent eye condition showed advantage in crossed dominance (0), while incongruent eye condition showed advantage in ipsilateral dominance (1). Performance was generally better (lower times) in ipsilateral dominance conditions across all phases.

**Figure 6:**
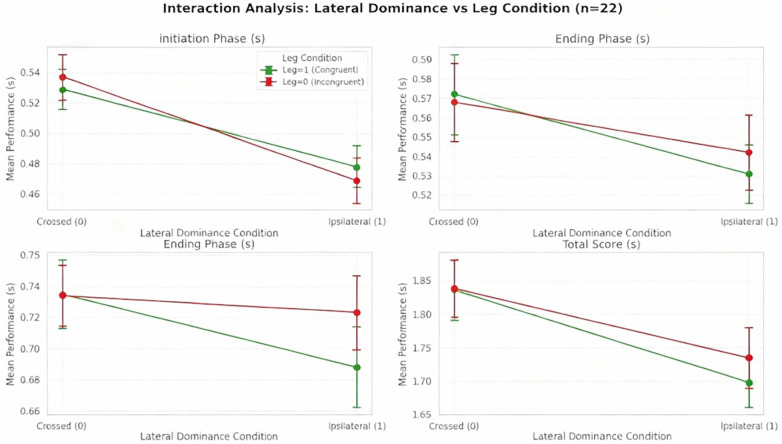
Interaction between lateral dominance and leg congruence on performance metrics. Performance times (mean ± SEM, *n* = 22) during **(A)** initiation phase, **(B)** turning phase, **(C)** ending phase, and **(D)** total performance. Green line: congruent leg condition (dominant leg aligned with movement direction). Red line: incongruent leg condition. A significant interaction pattern emerged where congruent leg condition showed advantage in crossed dominance (0), while incongruent leg condition showed advantage in ipsilateral dominance (1). The interaction effect was most prominent during the initiation phase.

Performance data across the three skiing phases showed the completion phase took the longest time, indicating that this phase accounts for the largest proportion of the total turn duration. In contrast, no significant main or interaction effects were observed in the steering phase. Notably, in the initiation phase, eye-turn consistency significantly influenced outcomes, with better performance observed during turns towards the non-dominant side (see Figure 5 and Figure 6).

In the initiation phase, the dominant eye model showed no interaction effect but revealed a main effect where ipsilateral dominance performance (0.474 ± 0.068s) was significantly better than crossed dominance (0.533 ± 0.066s, *t* = 2.5554, *P* < 0.05), with ipsilateral athletes performing better (*η*^2^ = 0.247). Eye-turn consistency also showed a significant main effect (*P* = 0.015, *η*^2^ = 0.009) (Table 2). For leg dominance, a significant interaction was found between dominant leg-turn consistency and lateral dominance (*η*^2^ = 0.009), with the best performance seen in ipsilateral dominance with inconsistent leg-turn direction (0.469 ± 0.071s) and the worst in crossed dominance with inconsistent leg-turn (0.537 ± 0.070s) (Table 3).

**Table 2:** Main and interaction effects of the mixed linear model for dominant eye.

Phase	Statistic	Crossed/ipsilateral dominance	Dominant eye	Crossed/ipsilateral dominance*dominant eye
Initiation Phase	β	0.058	−0.010	0.002
	F	6.886	5.996	0.062
	t	2.554	−1.908	0.249
	P	0.016*	0.015*	0.803
	95% CI	[0.011, 0.105]	[−0.019, 0.000]	[−0.012, 0.016]
	Partial *η*^2^	0.247	0.009	0.000
Turn Phase	β	0.037	0.011	−0.007
	F	1.247	2.894	0.598
	t	1.219	1.745	−0.767
	P	0.277	0.089	0.443
	95% CI	[−0.026, 0.100]	[−0.001, 0.024]	[−0.025, 0.011]
	Partial *η*^2^	0.056	0.005	0.001
Completion Phase	β	0.045	0.035	−0.033
	F	0.476	17.667	13.942
	t	1.081	5.612	−3.734
	P	0.498	0.000**	0.021*
	95% CI	[−0.042, 0.131]	[0.023, 0.047]	[−0.050, −0.016]
	Partial *η*^2^	0.022	0.027	0.021
Total Time per Turn	β	0.140	0.037	−0.038
	F	2.364	5.167	6.059
	t	1.771	3.348	−2.461
	P	0.140	0.023*	0.014*
	95% CI	[−0.033, 0.304]	[0.009, 0.057]	[−0.073, −0.011]
	Partial *η*^2^	0.104	0.008	0.009

*p < 0.05.

**p < 0.01.

**Table 3:** Main and interaction effects of the mixed linear model for dominant leg.

Phase	Statistic	Crossed/ipsilateral dominance	Dominant leg	Crossed/ipsilateral dominance*dominant leg
Initiation Phase	β	0.050	−0.010	0.017
	F	6.886	0.062	5.996
	t	2.212	−1.908	2.449
	P	0.016*	0.803	0.015*
	95% CI	[0.003, 0.097]	[−0.019, 0.000]	[0.003, 0.031]
	Partial *η*^2^	0.247	0.000	0.009
Turn Phase	β	0.041	0.011	−0.015
	F	1.247	0.598	2.894
	t	1.360	1.745	−1.701
	P	0.277	0.443	0.089
	95% CI	[−0.022, 0.104]	[−0.001, 0.024]	[−0.033, 0.002]
	Partial *η*^2^	0.056	0.001	0.005
Completion Phase	β	0.047	0.035	−0.037
	F	0.476	13.942	17.677
	t	1.131	5.612	−4.203
	P	0.498	0.000**	0.000**
	95% CI	[−0.039, 0.131]	[0.023, 0.047]	[−0.054, −0.020]
	Partial *η*^2^	0.022	0.021	0.027
Total Time per Turn	β	0.138	0.037	−0.035
	F	2.364	6.059	5.167
	t	1.753	3.348	−2.273
	P	0.140	0.014*	0.023*
	95% CI	[−0.026, 0.302]	[0.015, 0.058]	[−0.065, −0.005]
	Partial *η*^2^	0.104	0.009	0.008

*p < 0.05.

**p < 0.01.

In the completion phase (Table 2 and Table 3), neither the eye nor leg models showed significant main effects for lateral dominance (*P* > 0.05). However, both dominant eye-turn and dominant leg-turn consistency were significant (*P* < 0.001), showing better performance when consistent with the turn direction (eye: 0.716 ± 0.103s; leg: 0.715 ± 0.113s). Both factors showed significant interaction effects with lateral dominance (*P* = 0.021; *P* < 0.001), with the best performance occurring when both eye/leg dominance and turn direction were consistent within the ipsilateral dominance group (Figure 5 and Figure 6).

Overall analysis revealed phase-specific effects of lateral dominance, with crossed/ipsilateral dominance mainly influencing the Initiation phase where ipsilateral dominance showed better performance with a large effect size (partial *η*^2^ = 0.247). The completion phase advantage for eye dominance and the stable significant effects of eye-turn consistency in both completion phase and total performance (*P* = 0.000, *P* = 0.023) suggest visual dominance plays a key role in movement execution and overall performance (Table 2 and Table 3).

## 4 Discussion

Although alpine skiing is a bilateral sport requiring balanced performance on both sides, many skiers demonstrate better performance on one side compared to the other. Coaches often find it difficult to precisely identify the causes of this technical asymmetry. Typically, they advise skiers to replicate movements from their better-performing side on the weaker side, but these methods prove ineffective. This leaves many athletes, including myself, significantly affected by technical asymmetry. This study confirms that lateral dominance (including eye dominance, leg dominance, and ipsilateral/crossed dominance combinations) is an important factor influencing bilateral performance asymmetry, though it is not the sole determining factor. The effects show phase-specific characteristics, and the interaction effects between eye-leg coordination patterns are significant. In line with the research hypothesis, eye dominance and crossed/ipsilateral dominance patterns align with the expected results. However, interestingly, the dominant leg's impact deviated from the hypothesis, showing contrary effects, which may suggest that the role of leg dominance in skiing performance operates differently than initially assumed. These findings address the limitations of previous studies that focused on single dominance factors.

### 4.1 Phase-specific effects of the dominant eye

Existing studies on eye dominance primarily focus on clinical fields, with limited exploration of its impact on sports performance (37). Relevant research more often examines eye-hand or eye-leg coordination in ipsilateral/crossed dominance contexts, finding that eye dominance typically enhances performance in cricket, shooting, and archery—outcomes closely linked to neural synergy (14, 21, 38). In daily life, people naturally rely on their dominant eye when aiming during activities like dart throwing or telescope use. Multiple studies thus confirm that eye dominance appears to improve sports performance. However, this study discovered that athletes demonstrate superior performance when their dominant eye-turn direction inconsistency, with this phenomenon occurring exclusively during the initiation phase. Some alpine skiers often perceive visual differences between gates on either side—some appear easier to capture visually than others.

This novel finding may be explained by the initiation phase's requirement for rapid gate recognition and trajectory planning, which demands high visual-neural synergy (39). Similar to aiming in archery or tracking tennis ball trajectories for anticipation, consider this scenario: when an athlete their dominant eye-turn consistency (e.g., initiating a right turn with right eye dominance), their dominant eye becomes positioned uphill. This placement risks visual obstruction by facial anatomy or equipment, creating blind spots that delay visual information acquisition. In fact, when only one eye is used, the brain tends to prioritize information from the dominant eye, suppressing or omitting input from the non-dominant eye at the center of the visual field (26). Consequently, the brain requires extra time to integrate remaining visual cues, potentially triggering compensatory head or body rotation for better gate alignment. Once this alignment is achieved, the optimal edging timing may be missed, reducing movement precision (40–42). Similarly, in tennis, when positioned on their dominant eye-turn consistency but receiving a shot to their non-dominant side, players may need to rotate their head or body to align their dominant eye with the ball for targeting. Conversely, when turning toward the, the dominant eye remains outward with an unobstructed view, enabling efficient processing of distant gate information and shortening decision-making time during initiation (22, 38) frequently overlook the impact of visual-neural synergy during initiation phases, focusing solely on movement precision, which contributes to slow progress in performance enhancement.

Therefore, both general conditioning and sport-specific training cycles should incorporate specialized visual coordination exercises. These should enhance visual information processing efficiency across different skiing postures, particularly during initiation phases and when turning toward the dominant eye-turn consistency. By strengthening visual compensation mechanisms and neural integration capabilities, negative impacts can be reduced to improve sports performance.

### 4.2 Mechanisms of difference between ipsilateral and crossed dominance

Results indicate that athletes with ipsilateral dominance demonstrated better performance across all three phases and in the overall time. A significant interaction effect was observed between dominance type (crossed/ipsilateral) and both dominant eye and dominant leg in the overall performance, confirming that these factors influence each other. Although the dominant eye had a significant main effect in the initiation phase of the skiing run, this was not observed in the other two phases or in the total time, further supporting the phase-specific nature of alpine skiing. Many studies have shown that in precision aiming sports such as shooting and archery, as well as in soccer penalty kicks, athletes with ipsilateral dominance tend to perform better (9). In contrast, athletes with crossed dominance excel in interceptive sports such as tennis and baseball batting. The present study, however, found that ipsilaterally dominant athletes achieved superior performance.

#### 4.2.1 Ipsilateral dominance

Ipsilateral dominance refers to a configuration where the athlete's dominant eye and dominant leg are on the same side. This type of dominance is particularly beneficial in sports that require high levels of eye–leg coordination. In the final phase of a skiing turn, athletes must maintain speed while preparing for the upcoming weight shift (33, 43). This process involves two key actions: first, observing the position and condition of the next gate—or even the one after—to visually plan the upcoming trajectory; and second, initiating the weight transfer to gradually flatten the ski from its edged position (44). Due to the high frequency of turns in slalom skiing, weight shifts must be executed rapidly, placing extreme demands on instantaneous explosive strength and eccentric muscle control. During the initial part of the transition—toward the end of the turn completion phase—the athlete must not only counteract the resistance generated by the ski cutting across the snow but also manage the centrifugal force resulting from the change in direction (33, 45).

If an athlete has ipsilateral dominance — for example, with both the dominant eye and dominant leg on the right side — the mechanism may involve the direct transmission of visual information about gate location perceived by the right eye to the motor control centers governing the right leg. This close coordination between visual input and motor execution likely enhances the athlete's ability to respond quickly and accurately, as the brain processes both visual and motor commands within the same hemisphere, minimizing delays and optimizing performance. This streamlined pathway facilitates quicker and more stable movement execution, particularly during turns toward the dominant side. The neural mechanisms underlying this advantage can be explained from the perspective of interhemispheric cooperation and information-processing efficiency (46). The hypothesis is that when one side of the brain (e.g., the left) processes both visual input (e.g., from the right eye) and motor control (e.g., for the right leg), there is less need for communication between the brain's two halves. This reduces delay and saves energy (47).

#### 4.2.2 Crossed dominance

In contrast, the situation is more complex for athletes with crossed dominance. For example, if the dominant eye is on the right side while the dominant leg is on the left, the brain must transmit visual signals regarding gate information across the corpus callosum to engage the dominant leg on the opposite side (48). The proposed mechanism is that when visual signals from one eye (e.g., the left, processed in the right hemisphere) and motor commands for the limb on the same side (e.g., the left leg, controlled by the right hemisphere) must be integrated, it may require interhemispheric transfer. This transfer could introduce a neural delay and increase the cost of coordination, potentially resulting in reduced movement accuracy. Consequently, this might explain why such actions can sometimes appear slightly slower or less synchronized (49, 50).

However, with long-term training, this requirement for cross-lateral coordination can compel athletes to develop skills on both sides. Although performance on one side may not be as stable as that of ipsilaterally dominant athletes, their overall movement may become more symmetrical bilaterally (51).

From a practical skiing perspective, another possibility arises. Previous biomechanical studies have shown that during carved or parallel turns, pressure distribution and ground reaction forces between inside and outside legs vary significantly, and that inside leg sometimes carries substantial load during the turn transition or specific turn elements (52, 53). Based on this, it is plausible that when the inside leg is also the non-dominant leg (i.e., not the skier's stronger/more stable leg), its ability to resist centrifugal forces and provide stable support may be compromised. This could contribute to reduced movement efficiency or stability during turns, especially under high speed or tight turn conditions. This can force the skier to adopt a safer but slower upper-body crossing technique. More critically, insufficient force production from the non-dominant leg may lead to overcompensation by the dominant leg. During weight transfer between legs, this imbalance can cause the skier's center of mass to become stuck midway or toward the uphill side. This phenomenon often results in sustained rebound of the ski, increased pressure and friction between the ski edge and the snow, a longer trajectory, and ultimately impaired competitive performance.

The underlying mechanism is hypothesized as follows: in slalom skiing, this cross-dominant processing may easily induce cognitive overload, which could exacerbate delays during the turn completion phase. Even after motor commands are issued, the required interhemispheric coordination might cause deviations in the timing and magnitude of force application by the dominant leg. These deviations are thought to be particularly amplified during rapid balance recovery and weight adjustment, ultimately compromising movement fluency and precision.

### 4.3 Practical applications for training

The findings of this study and the preceding analysis suggest that lateral dominance plays a role in athletic performance. Therefore, targeted training is essential to enhance its positive effects and mitigate the negative impacts of technical asymmetry. Current physical training aimed at addressing lateral dominance often focuses primarily on strengthening the non-dominant side. However, this approach is overly simplistic and does not meet the specific demands of alpine skiing, nor does it fully address the implications of lateral dominance.

Lateral dominance—such as dominant eye, dominant leg, and crossed or ipsilateral dominance patterns—has an impact on performance. Previous research on motor lateralization and interlimb transfer has shown that unilateral training can lead to functional improvements in the contralateral limb, and that brain hemispheres contribute differently to dynamic and stabilizing control. In another sport, laterality-specific training of the non-dominant leg improved coordination and spatial cognition (54). Based on these findings, it is plausible that long-term, balanced training (or deliberate cross-lateral practice) in skiing might help athletes develop more symmetrical bilateral skiing performance, even if one side remains less stable than the ipsilaterally dominant configuration. Thus, training should be designed to leverage its benefits while minimizing the drawbacks of technical imbalances. In alpine slalom skiing, the high density of gates demands a rapid turning rhythm. Athletes must maintain stable visual focus on the gates while executing automated turning movements at high speeds.

Under these conditions, strength training alone is insufficient to meet event-specific demands. Integrated visual-motor coordination training becomes particularly important. To address this, dual-task or dual-focus drills can be incorporated into physical conditioning (55). For example, athletes can perform posture transitions (e.g., sitting–kneeling–standing) on a balance pad or yoga ball while simultaneously engaging in a ball-tossing task with the upper limbs. This can be further upgraded to a “triple-task” protocol: adding elastic band arm movements (simulating gate contact during turns) while visually tracking two randomly moving tennis balls and maintaining continuous scanning. Additional progressions may include replacing the tennis balls with cards displaying the word “green” printed in yellow ink, requiring the athlete to name the color rather than read the word. Such exercises simulate the complex environmental demands of skiing by integrating multiple tasks, thereby enhancing neural coordination and automaticity under unstable conditions. The variation in task design not only improves visual-cognitive function but also significantly boosts reaction speed and decision-making.

These methods can be integrated into a functional training program, implemented 2–3 times per week, with each session comprising 4 sets. Long-term adoption of such training can effectively enhance neuromuscular coordination and visual integration, improving overall performance in complex environments.

In practice, athletes with different dominance profiles require individualized training programs. Those with ipsilateral dominance—where the dominant eye and limb are on the same side—often exhibit strong-side reliance and weak-side deficiencies (56). Training for these athletes should emphasize bilateral synchronization and avoid one-sided dependency. Off-snow training may involve lateral hurdle jumps synchronized with a metronome. On-snow drills may include single-leg gate passing: for example, setting a linear series of 8–10 closely spaced gates on the left side, which the athlete navigates using only the left leg, then repeating on the right. This can be progressed by incorporating slalom gates with moderate turning angles, gradually improving inter-limb coordination and stability under varied rhythmic demands.

In contrast, athletes with crossed dominance should focus on enhancing overall coordination and establishing automated movement patterns to reduce the cognitive load associated with cross-lateral visual compensation. During on-snow gate training, short poles can be placed on both sides of the course, with additional slalom poles angled outward placed inside the line of the gates to restrict upper-body rotation. Gate spacing should initially match the turning radius of the skis and be gradually reduced. Mid-course, 3–4 serpentine gates can be added to increase movement complexity. Angled poles inside the line may also be used to limit excessive upper-body swing. This approach not improves bilateral movement rhythm but also enhances neural control of complex motor skills.

Through such differentiated training, ipsilaterally dominant athletes can develop more balanced motor competence, while crossed-dominant athletes can form more stable and efficient automated technical patterns under high-intensity conditions.

### 4.4 Limitations

This study utilized a ski simulator to investigate the impact of lateral dominance on alpine skiing performance during slalom turns. Although this study's use of a ski simulator, while offering controlled conditions for investigating lateral dominance, does not fully replicate the complexities of on-snow skiing, particularly regarding snow conditions and slope variability. Although simulator-based kinematics are strongly correlated with real-world skiing (57), future studies should validate these findings under on-snow conditions. The small sample size in this study, with 22 athletes, limits the generalizability of the results, reflecting the difficulties in recruiting high-level skiers, particularly in the national-elite athlete groups. This sample size is consistent with similar studies in alpine ski racing. For example, one study used 23 athletes (58), while another reported a sample of 20 athletes to analyze performance factors in elite alpine skiers (25). Penitente et al. (2) also noted the difficulties associated with small sample sizes in their work with elite athletes, especially when the athletes come from highly specific training backgrounds (2). But caution is needed when extrapolating to a broader population. Despite these limitations, the study provides valuable insights into the role of lateral dominance in skiing performance. Additionally, all athletes were affiliated with the Chinese national team, and their specific training and ski racing contexts may have influenced the results. Future research with larger and more diverse samples is needed to further validate these findings and assess their broader applicability.

## 5 Conclusion

This study demonstrates that the performance of alpine ski racers is significantly influenced by lateral dominance. The dominant eye affects skiing efficiency due to differences in visual field obstruction. Athletes with ipsilateral dominance (dominant eye and leg on the same side) performed better in initiation and completion phases, which can be attributed to more efficient neural integration of visual and motor information within the same hemisphere. In contrast, athletes with crossed dominance showed poorer performance when turning toward their dominant leg-turn consistency, likely due to interhemispheric transmission delays. Technical asymmetry may also be associated with lateral dominance. It is recommended that training programs incorporate bilateral symmetry exercises and sport-specific drills for the non-dominant side, aiming to enhance synergistic efficiency in ipsilaterally dominant athletes and improve cross-body integration in those with crossed dominance. These approaches can reduce the risk of technical asymmetry and ultimately enhance competitive performance.ution and overall performance.

## Data Availability

The raw data supporting the conclusions of this article will be made available by the authors, without undue reservation.
